# Feeding decision-making among first generation Latinas living in non-metropolitan and small metro areas

**DOI:** 10.1371/journal.pone.0213442

**Published:** 2019-03-18

**Authors:** Maria Pineros-Leano, Karen Tabb, Janet Liechty, Yvette Castañeda, Melissa Williams

**Affiliations:** 1 School of Social Work, Boston College, Boston, Massachusetts, United States of America; 2 Illinois Transdisciplinary Obesity Prevention Program (I-TOPP), Urbana, Illinois, United States of America; 3 School of Social Work, University of Illinois, Urbana, Illinois, United States of America; 4 College of Medicine at Urbana, University of Illinois, Urbana, Illinois, United States of America; 5 Carle-Illinois College of Medicine, University of Illinois, Urbana, Illinois, United States of America; 6 Department of Kinesiology and Community Health, University of Illinois, Urbana, Illinois, United States of America; 7 Office of Minority Student Affairs, University of Illinois, Champaign, Illinois, United States of America; Tulane University School of Public Health and Tropical Medicine, UNITED STATES

## Abstract

**Background:**

Worldwide, overweight and obesity rates have more than tripled over the past three decades. Overweight and obesity rates are particularly high among Latinos. In order to determine some of the potential reasons, it is imperative to investigate how first-generation Latina mothers living in non-metropolitan and small metro areas decide how and what to feed their children. Using the Socio-Ecological Model, this study aimed to understand how Latina immigrant mothers make feeding decisions for their children.

**Methods:**

A total of 29 semi-structured interviews were conducted with a purposive sample of immigrant mothers from Latin American countries whose preschoolers were enrolled in a Women, Infant, and Children supplemental nutrition program located in non-metropolitan and small metro areas. All interviews were recorded and transcribed verbatim in Spanish, and analyzed by a bilingual team.

**Results:**

Multi-stage qualitative analysis was employed to analyze the data. Nineteen participants originated from Mexico, four from Central America, and six from South America. Five themes emerged that helped illuminate mother’s decision-making around feeding choices: 1) culture as all-encompassing, 2) location and access to fresh and traditional foods, 3) disjunction between health provider advice and cultural knowledge 4) responsiveness to family needs and wants as determinants of food choices, 5) intrapersonal conflict stemming from childhood poverty and food insufficiency.

**Conclusion:**

Findings suggest that Latina immigrant mothers engage in a difficult and even conflicting process when deciding how to feed their children. Future interventions should focus on implementing hands-on activities that can help consolidate, promote, and encourage healthy feeding choices.

## Introduction

The World Health Organization has declared the “global obesity epidemic” as one of the most pressing public health concerns [1]. Globally, overweight and obesity have tripled in the last 30 years [1]. In the United States (U.S.) the rates of obesity are higher among Latinos than any other racial/ethnic group [2]. Seventy-seven percent of Latinos in the U.S. over the age of 20 years are overweight or obese, compared to 67% of their White counterparts. Among children, Latinos also have the highest prevalence rate (38.9%) of any other racial/ethnic group [2]. For this reason, it is necessary to investigate the underlying factors for the high prevalence of obesity among Latinos.

A particular characteristic of the Latino population in the U.S. is that almost half of adult Latinos (47.9%) are born in a foreign country or territory [3]. Nativity is an important factor to consider along with health as it has been previously shown that first-generation, less acculturated Latino immigrants have better health outcomes compared to their more acculturated counterparts [4–6]. The health advantage of more recent immigrants has been termed the immigrant health paradox and it extends to different health areas, including obesity [7–10]. For instance, a cross-sectional study of 6,421 immigrants residing in the U.S. demonstrated that earlier age of arrival and greater time spent in the U.S. were associated with increased rates of overweight and obesity [8]. Other studies have demonstrated similar trends, including a systematic review of the association between duration of residence in the U.S. and body mass index (BMI) [10]. The review study synthesized findings from 15 studies conducted in the U.S. and found that 14 of the studies demonstrated a positive association between BMI and years of residence in the U.S. and this relationship was particularly strong among Latino immigrants [10].

Reasons behind the increase in overweight and obesity among Latino immigrants include changes in nutrition and diet [8, 11–14]. A cross-sectional study (n = 2,132) demonstrated that the more time women spend in the U.S., the more their diet changed [12]. A qualitative study (n = 51) also found unhealthy changes in diet among Latina immigrant women with moderate levels of acculturation, including less consumption of fresh fruits and vegetables and more consumption of fast food [15]. Other studies have suggested similar results, indicating that more assimilated Latino immigrants consume more fast foods, added fats, sugar, fatty snacks and less fruits, vegetables, grains, fiber, and legumes compared to less acculturated Latinos [13, 14]. Overall, these previous studies have found that after migration, people change their feeding practices, particularly women. Keeping in mind that women are more likely to be in charge of cooking meals at home [16], and that preschool-aged children eat most of their meals at home [17], it is necessary to understand how first-generation Latina mothers decide to feed their children.

Prior research investigating maternal decision-making around food choices has primarily been conducted in metropolitan cities (e.g., [16, 18–21]). For example, a study conducted in San Diego with 41 Latina mothers investigated their attitudes around feeding choices and found, not surprisingly, that mothers felt their main responsibility was to feed their children well [16]. Another qualitative study conducted focus groups and in-depth interviews in Boston with Latina women (n = 51; 21). The study found that maternal feeding practices were highly influenced by the grandmothers’ opinions about the weight of the child. Although there are a few studies investigating decision-making around feeding strategies in metropolitan cities, there is a dearth of research in non-metropolitan and small metro areas. It is important to understand feeding choices in these areas because maternal choices may be more constrained, and contributors at different levels of social ecology may be operating differently to influence decision-making.

## Theoretical framework

The Socio-ecological Model [22, 23] served as a guide to understand the complex processes that take place when mothers decide how to feed their children. This model suggests that people’s health behaviors are influenced by multiple levels that include intra-personal and interpersonal factors, institutional factors, community factors and public policy factors [22, 23]. This model provides a lens to examine and better understand the layers of competing tensions that Latina immigrant mothers may experience at different levels of their social ecology when making feeding decisions.

To address the aforementioned gaps, the purpose of this study was to explore and begin to characterize the following: How do Latina immigrant mothers living in non-metropolitan and small metro communities make decisions about how and what to feed their children?

## Methods

### Sample

Participants for this study were recruited from the Women, Infant, and Children (WIC) supplemental nutrition program. The inclusion criteria were: being a mother, having a child between 1 month and 5 years, being first generation Latina, being willing to be measured for weight and height, being older than 18 years of age, and agreeing to participate in the study.

### Recruitment strategy

We used a purposive sampling strategy that consisted of four different methods. The first method used information from a larger study conducted in 2012 with mothers who were part of the WIC program. As part of this study some participants agreed to be contacted for future studies, and 57 Latina women who indicated willingness to participate in future studies were contacted; of these, only 18 could be reached. Four of them were not interested in participating in the study, five were not eligible, and nine were eligible and accepted to participate. Out of the 39 mothers that could not be reached, 17 phone numbers were out of service, 7 had changed their number, and 15 did not answer the calls.

The second recruitment method was face to face. The first and last authors recruited 13 mothers from the WIC office waiting room. The third recruitment strategy was the use of flyers placed around the WIC office with information in Spanish language about the study. Only two mothers called and expressed their interest in participating from these flyers. A final recruitment strategy was participant referral. After the interview, each mother received two flyers to refer friends to the study. A total of five participants were recruited through this method. The final sample included 29 mothers.

### Data collection

Data were derived from twenty-nine in-depth interviews conducted from May through September, 2015. The first author (MPL), a female native Spanish speaker, conducted all the interviews and collected field notes during and after the interview. Prior to the beginning of the study only two participants had had previous interactions with the interviewer from previous qualitative studies.

**Fig 1:**
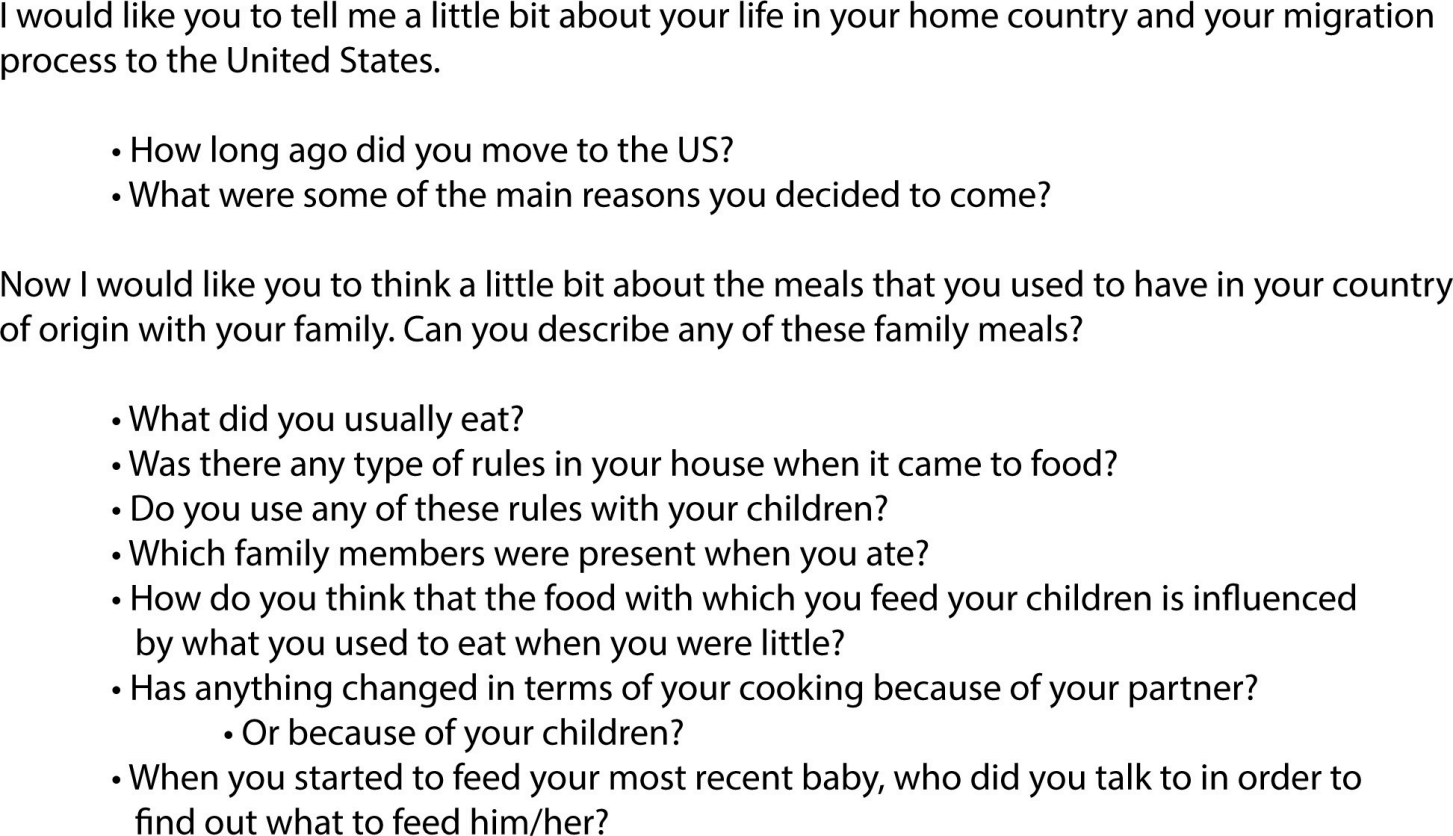
Example of semi-structured interview questions.

All the interviews were conducted in Spanish and they asked participants for a retrospective account of their eating behaviors and family feeding practices while growing up and also after migration to the U.S. (Fig 1). The questions were always asked in the same order to all participants and only one interview by participant was conducted. Given time constraints, neither repeat interviews nor member checking were conducted. Direct questions around the weight status of the child were not included in the questionnaire given the sensitive nature of this question and to avoid priming participants to focus on weight status, unless this was a concern raised by the mothers themselves. It is important to note that a few mothers did mention the weight status of their children during the interview.

All the interviews were conducted at a location of the participants’ choosing. Most of the interviews were conducted in the homes of the participants while their child/children were present. In order to make sure that the participant could fully participate in the interview, child care was provided by a research assistant. In two instances the participant’s partners were sitting close by but did not participate in the interviews. Before the beginning of the interview, the first author read the consent form to the participants and obtained their written consent to participate in the study. The meeting consisted of a questionnaire on demographics, acculturation, mental health, and feeding practices; followed by data collection of mother’s height and weight; and ultimately an in-depth interview lasting an average of 32 minutes. All the interviews were audio-recorded with permission from the participant. The overall protocol, including the questionnaires, anthropometric data collection, and interview lasted approximately two hours. A $40 gift card was given to participants as remuneration for their time. All procedures were approved by the Institutional Review Board at the University of Illinois at Urbana-Champaign.

### Data analysis

All the interviews were transcribed verbatim by two bilingual researchers (MPL and MW). Relevant quotes were then translated into English. Consistent with a grounded theory approach, we allowed the themes to emerge from the data employing a multi-stage qualitative coding analysis that consisted of four stages [24]. For the first stage we used initial coding, where we read through all the interviews and identified initial codes, reaching data saturation (i.e., no additional codes) at 23 interviews. Once we completed initial coding on all the interviews, we selected the most salient codes and assembled a codebook. After the codebook was in place we began a second wave of axial coding, which consisted of recoding the interviews based on the newly developed codebook using QSR International's NVIVO 11 Software. During the third stage we developed categories from the codes based on families of codes [24]. The fourth stage required “themeing” the data where three coders (MPL, YC, and MW) grouped the previously developed categories, found relationships, and selected the most salient themes [24]. The coders’ bilingual and bicultural experience offered a unique lens through which the experiences of first-generation Latina mothers were understood and analyzed.

To enhance trustworthiness and assure reliability and validity of qualitative data, we used analyst triangulation and inquiry audit [25, 26]. Analyst triangulation consisted of comparing the codes developed by three bilingual and bicultural coders to encourage multiples points of view [26]. Inquiry audits consisted of having two senior researchers (KT and JML), not involved in the analysis stage, to oversee the final themes and examine the overall process of inquiry [27].

## Results

### Sample description

Nineteen participants were from Mexico, four from Central America, and six were from South America (Table 1). All the participants lived in non-metropolitan and small metro areas in a county with fewer than 250,000 residents. Participants lived within a 25 mile radius of the county WIC office. Most (n = 25) women were married or living with a partner. The average time spent in the U.S. was 10 years (standard deviation (SD) = 5.9). The average BMI of the mothers was 28.8 (SD = 4.7), and average age was 32 years (SD = 4.9).

**Table 1:** Demographic characteristics of participants.

Characteristic	Mean	SD	%	n
Education				
Less than high school			51.7	15
High school			13.8	4
Some college			17.2	5
College and more			17.2	5
Employment				
Not employed			65.5	19
Employed			33.3	10
Marital status				
Married/cohabiting			82.8	24
Single/divorced			16.7	5
Age	31.9	4.9		
Time spent in the US (years)	10.1	5.9		
BMI	28.8	4.7		
N =				29

*Note*: Body mass index (BMI). Standard deviation (SD)

### Themes

Overall, the results showed that our participants faced the tension of having to decide between keeping their traditional background and adapting to a new context. In many instances, the mothers had to navigate between two or more internal values or tensions. Thematic analysis uncovered five themes that illustrate internal tensions and competing influences on maternal decision making about what and how to feed their children. These different influences were organized into the five levels of the Socio-Ecological Model ranging from the most macro-level (i.e. culture) to the most micro-level (i.e. individual). Table 2 presents a summary of the different levels of the Socio-Ecological Model, the identified theme from the study, and an example of each theme. The themes that we identified were the following: 1) Culture as all-encompassing, 2) Location and access to fresh and traditional foods, 3) Disjunction between health provider advice and cultural knowledge, 4) Responsiveness to family needs and wants as determinants of food choices, and 5) Intrapersonal conflict stemming from childhood poverty and food insufficiency. Next, we provide a description of each theme with exemplary quotes from the participants. All the names have been changed for confidentiality purposes.

**Table 2:** Themes identified at each level of the Socio-Ecological Model.

Level	Relevant Theme(s)	Examples
Culture	Culture as all-encompassing	Cultural influence of foods prepared in host country Cultural traditions around feeding
Country/policy	Location and access to fresh and traditional foods	Availability and food access to fresh produce easier in country of origin
	Disjunction between health provider advice and cultural knowledge	Discrepant advice between health providers in the US and country of origin WIC nutrition education needs to be more culturally sensitive
Community	Location and access to fresh and traditional foods	Lack of familiar produce Change of diet after migration to adapt to lack of fresh produce
Clan/ Family	Responsiveness to family needs and wants as determinants of food choices	Child food preferences Ensuring family wellbeing
Individual	Intrapersonal conflict stemming from childhood poverty and food insufficiency	Maternal history of food insecurity

#### Culture as all-encompassing

Throughout the majority of the interviews it became clear that the main driver of decision-making about food choices for their children was cultural tradition. One mother, referring to the family meals she prepared, said: “We bring our tradition in our blood” (Natalia, age 28). This statement was similar to the opinion of 20 other participants, who mentioned that the food they prepared was similar to the food they ate growing up.

*“I make them enchiladas*, *rice with chicken*. *Similar food to the one in Honduras*.*” (Daniela*, *age 36)*

Sixteen participants also mentioned that although they make similar foods to those they had when growing up, they try to make them healthier by increasing the vegetable content and avoiding cooking with high fats like lard. For example:

*“I teach them to eat fruits and vegetables and also different foods*, *not only Mexican; more international [foods]” (Valeria*, *age 40)*.

*“Over there [in Mexico] we used to cook with lard*, *but I know it’s not healthy*. *I try for [the food] to be similar to the one from Mexico*, *but I don’t cook with lard” (Julia*, *age 40)*.

Along with food, there were other traditional cultural practices around feeding that continued to be reinforced. For example, 13 participants mentioned that growing up they were forced to finish all the food that was on their plates, a practice enforced by some of the mothers with their own children. However, the cultural reason for enforcing this rule was mostly to avoid wasting food.

*“I always tell them to serve themselves whatever they are going to eat and also not to serve that much*. *I prefer them to serve little at first and then go for more than serving a lot and then throw it out” (Daniela*, *age 36)*.

Not all participants mentioned asking their children to eat all the food that was on their plates. Five mothers reported that they no longer enforced this rule because they learned from health providers in the U.S. that it was not healthy. For example, a participant said:

*“…I was forced to eat*. *And that’s what I find weird with my child because the doctor says that he has to eat whatever he wants from the healthy food that I present… So we don’t force him to eat” (Bianca*, *age 32)*.

The participants indicated that there is tension between keeping cultural traditions alive and adapting to new recommendations. On the one hand, participants reported wanting to remain tied to their cultures by preparing traditional dishes and eating similar foods to those from their country of origin. On the other hand, they were also willing to change some practices around food and feeding to promote the health of their children.

#### Location and access to fresh and traditional foods

Eighteen participants articulated that fresh food was better for the family since it provided more nutrients than fast or processed foods. Moreover, 14 participants argued that fresh food had a better taste. However, eight participants mentioned that it was more difficult to access fresh food in the U.S. compared to their country of origin where fresh fruits and vegetables were readily available at a lower cost and within walking distance of their home. The participants mentioned that this was particularly the case when living in a non-metropolitan/small metro Midwestern city, where car access is necessary to reach a supermarket or a convenient store. For instance, Claudia described that it was easier for her to go grocery shopping back in Mexico than in the U.S:

*“To me it’s easier going grocery shopping there [in Mexico] than here… Because over there you’re always walking… Here everyone needs to know how to drive and I don’t drive*, *so it’s more difficult” (Claudia*, *age 34)*.

Eleven participants felt that after migrating to the U.S. they found it difficult to get used to the lack of fresh food available and expressed having a difficult time accessing fresh fruits and vegetables whereby these foods may be available all year round, providing greater access in the country of origin. After immigrating to non-metropolitan and small metro areas, participants felt their choices were limited to seasonally available foods, which limited fruit and vegetable consumption.

*“Where we used to live we used to harvest from the land… Here is not the same*, *it [the food available] depends on the season” (Elizabeth*, *age 23)*.

*“…Sometimes here there aren’t [certain foods]*, *especially here in [non-metropolitan area]*, *there are places where you can buy but if they don’t have them*, *you have to travel all the way to Chicago” (Lucia*, *age 34)*.

Given the lack of access to fresh produce in the area, some participants started changing their eating patterns and adapting to the foods that were available, even if that meant serving their children less healthy processed foods.

*“For example*, *we eat a different type of food in Mexico*. *We don’t eat what we eat here*. *Here we eat too much bread and canned [food]*. *Over there is more organic food*, *fresh” (Paula*, *age 29)*.

#### Disjunction between health provider advice and cultural knowledge

When prompted about how the participants decided to feed their children and who they obtained information from, 24 participants said that their information came from WIC providers, family members, and pediatricians from their country of origin. Through these conversations, it became clear that some of the knowledge they had previously obtained conflicted with the information they obtained from WIC. For instance, a mother mentioned:

*“… The baby eats one egg per day*. *Sometimes I get worried because they [the doctors] have told me that I could only give him egg twice per week… But in Colombia they told me that eggs are important*. *I don’t know who to listen to…” (Sofia*, *age 35)*.

A couple of participants also mentioned that although most of the information obtained from WIC was valuable and helpful, some of it could be more culturally sensitive. Three mothers expressed they had little exposure to some of the foods that WIC provided and they also expressed uncertainty on how to prepare those foods. Also, there seemed to be consensus among participants on wanting to obtain more hands-on training on how to cook/prepare the foods they obtained from WIC. A participant exemplified this by saying:

*“Do you know what would be really nice with the things that WIC provides? That there were a space for training the moms where they learn how to cook those foods*. *For example*, *[*…*]* *I get the oat meal and I’m thinking I should not keep getting so much because I feel bad having it there and not using it*… *But [it would be great] if there were a training where they told me “look*, *you can make this with oat meal…” (Sofia*, *age 35)*.

Six other mothers also mentioned they wished WIC could give them examples of the nutritional properties of foods. The participants mentioned they were aware of the need to have a balanced meal; however, they were not always certain about the nutrients in the foods provided by WIC.

*“Lunch is our main meal*. *Usually*, *it includes rice*, *beans*, *meat*, *salad*, *and steamed vegetables…” (Rosa*, *age 30)*.

*“…they tell me to eat proteins and grains… but they don’t tell me what proteins are… they could give me more information about what proteins are and in which foods I can find them” (Valeria*, *age 40)*.

#### Responsiveness to family needs and wants as determinants of food choices

The majority of participants described their children’s health as an important determinant of food choice. Particularly, 16 participants mentioned that they changed their eating practices to healthier ones after having children and/or to help their children lose weight.

*“…because of mine and my child’s overweight status*, *especially the ten year old one*, *I try to cook healthy food for him*. *It’s not easy because […] children of his age like to eat*, *so I try to control him… if I do not control him*, *he eats whatever he wants and does not exercise… I basically changed the things I ate for them…” (Lucia*, *age 34)*.

*“When I was alone, I would not cook; I was eating out. Now that I have my children, I cook at home almost all the time” (Veronica, age 34)*.

Twelve participants also mentioned changes in eating practices after the onset of a health condition such as gestational diabetes. Onset of chronic disease served as a wakeup call that prompted many women to start implementing healthier behaviors, such as eating more fruits and vegetables, and increasing physical activity for themselves and their families. To address these health problems, many participants mentioned having to change from fatty and high sugar content food to healthier alternatives.

*“…They found I had [high] glucose during pregnancy*. *They put me on a diet and I only ate vegetables*, *they took away everything*. *Since I was craving sweets a lot*, *they took them away*, *so I could only eat vegetables… [It was] just high glucose but if I did not take care of myself*, *then it would become diabetes so I tried to eat healthier for my son” (Gabriela*, *age 31)*.

Many participants also described disagreeing with their children over food choices. Four mothers who had children attending daycare or school recounted that disagreements arose because she prepared traditional foods from her country of origin, such as soups and stews; however, the children -who had been exposed to more “Americanized” foods-, preferred foods such as pasta and pizza. A few participants described giving into the child’s desires and learning how to make the foods their children asked for.

*“I fight with my daughter every day… She likes processed foods better*. *Sometimes*, *when she goes to the store with me*, *she grabs pizza rolls*, *pizza*, *chicken nuggets*…” *(Helena*, *age 38)*.

*“Yes because [my daughter] sometimes asks me to make macaroni and cheese and that’s something I had never even tried before*. *So that’s something I make for her now”*. *(Claudia*, *age 34)*.

The participants mentioned that the needs of the family were important forces to decide what to cook. Many of them changed their feeding practices after they were diagnosed with a certain condition that could risk the wellbeing of their children. Other mothers made changes in their feeding practices after they became parents. In both circumstances, our participants tried to do the best they could to make sure their children would stay healthy. However, once children started being exposed to foods that were different to those cooked at home, they started having input on the choices their mothers were making. In some instances there was a tension created between the mother’s feeding choices and the child’s. Some mothers managed this tension by only providing traditional food and others resorted to learn to prepare the foods their children liked.

#### Intrapersonal conflict stemming from childhood poverty and food insufficiency

One aspect that became clear was that some of the participants had an internal conflict about the foods they should feed their children. Among eight of the participants, part of this conflict stemmed from the level of poverty and food insufficiency that many of the mothers experienced growing up. All our participants felt the need to give their children a better future. However, a more prosperous future entailed giving their children the ability to *choose* what to eat; which was something they lacked growing up. The following quote exemplifies the level of poverty that some participants struggled with growing up. After asking Beatriz what she ate growing up, she said:

*“…just salt and a little of water was what we ate [in Guatemala]*. *There weren’t many tortillas; we did not have [any]*, *at least not there*. *We wanted to eat but there was nothing” (Beatriz*, *age 25)*.

Participants wanted to make sure that their children could have more choices than they did growing up. Moreover, because they now have more means to provide their children with food, they felt it would be unfair not to let them *choose* or to force them to eat something they did not want to.

*“I come from a country where not all needs are met right? And here thank God they have yogurt*, *milk*, *cereal; they have the luxury of saying “I don’t want anymore”*. *Look*, *they have the luxury of saying “oh I don’t want this apple anymore” (Catalina*, *age 35)*.

The participants interviewed expressed the tensions they had when deciding what and how to feed their children. At the intrapersonal level, the tensions they felt were intrinsic and dated back to their childhood. Allowing their children to choose their foods might be judged by professionals and researchers as permissive if the context and reasons behind this “permissiveness” is not understood. However, it is necessary to bear in mind that in many instances, low-income immigrant populations have a long history of food insufficiency and they might give in into their children’s request because they want to provide them with the food and resources they lacked growing up.

## Discussion

This study explored how first-generation Latina mothers make feeding decisions when living in non-metropolitan and small metro areas. We found that mothers engage in a complex process when making feeding decisions for them and their children. The Socio-Ecological Model helped explain the different influences that participants had when making feeding decisions. These decisions ranged from the influence that culture has on feeding decisions, to the intrapersonal conflict that participants felt when having to decide what to feed their children. We discovered five themes related to determinants of mothers’ feeding choices for their children ranging from culture, the more macro-level of the socio-ecological model, to the individual, the most micro-level of the model: 1) culture as all-encompassing, 2) location and access to fresh and traditional foods, 3) disjunction between health provider advice and cultural knowledge 4) Responsiveness to family needs and wants as determinants of food choices, 5) intrapersonal conflict stemming from childhood poverty and food insufficiency. Connections to the literature and implications the themes are discussed below.

### Culture as all-encompassing

Culture has been previously found to be the main driver of feeding choices among first-generation Latinas [18, 28]. A qualitative study of Mexican-American women (n = 21) found that culture and tradition were highly influential on the foods the mothers chose to cook for their families [28]. Our study also found that culture was a strong determinant of the foods that mothers chose to feed their children with. Similar to our findings, Smith et al., [28] found that women were less likely to cook with lard than with oil. This is an important finding because it suggests that there is a cultural shift that is taking place among Latina mothers. It is possible that this change has been influenced by educational campaigns from health and public health providers. Thus, it is important to continue reinforcing this information among Latina immigrant women.

A novel finding from this study was that although culture is a defining factor of feeding choices and practices among Latina immigrant mothers, they are highly receptive to new messages. For instance, a few mothers in our study mentioned that given all the information they had received from WIC and their pediatricians, they decided not to pressure their children to eat everything that was on their plates. This finding suggests that public health campaigns addressing unhealthy feeding choices and practices can be beneficial. Latina immigrant mothers are listening and adopting some of these messages to improve the wellbeing of their children. We recommend health providers continue encouraging mothers to avoid pressuring their children to eat and finish all the food on their plates [29]. We also recommend that this encouragement be provided with an explanation of satiety cues and the health benefits of listening to them.

### Location and access to fresh and traditional foods

Participants felt that accessing fresh produce was much more difficult in non-metropolitan and small metro communities than it was in their country of origin. Some women argued that access was more difficult because the distances were greater so they always had to drive. For some other participants, access was more difficult because they could not harvest their own produce as they did in their country of origin. Similar results have been found in previous research [30, 31]. A study among women from Brazil, Haiti, and Central and South American countries found that participants felt they had more access to fresh food in their home countries compared to the US [30]. This finding suggests additional constraints on feeding choices that may be specific to non-metropolitan and small metro communities where access to supermarkets requires driving long distances [32].

The perception of limited access to fresh foods is an important barrier to address, particularly in non-metropolitan and small metro areas with growing immigrant populations. Several strategies may increase access to fresh foods in these particular settings. First, WIC and other health providers can host a small version of the Farmer’s Market at their facilities. Second, researchers and practitioners can help develop community gardens in predominantly Latino immigrant communities to increase access to fresh produce and also to help Latino immigrants remember their country of origin, where they could harvest their own foods. Third, practitioners and researchers can work alongside convenience/corner stores to find ways to carry fresh produce. Often, Latino immigrants have difficulty accessing fresh produce because they live far from large supermarkets; however, they might live close to convenience/corner stores and encouraging these stores to carry fresh produce could address this barrier. Overall, it was clear that our participants missed the freshness and quick access they had to fresh produce in their country of origin; thus, facilitating access or creating easily accessible spaces would help address this need.

### Disjunction between health provider advice and cultural knowledge

It was clear from our findings that Latina immigrant women already have a strong knowledge system on which to build. They have obtained previous nutritional information either from pediatricians in their country of origin or their family members. Similarly, a qualitative study in San Diego found that Latina mothers were knowledgeable about healthy foods and healthy cooking strategies, which they had learned from family members [16]. Throughout our interviews it also became clear that participants had a strong knowledge base about the foods that were nutritious and those that were not. However, they had difficulty accessing the healthy foods. Since Latina immigrant women already know which foods are nutritious, it is important to move beyond an approach based exclusively on education. Instead, it would be useful to promote campaigns that are culturally sensitive and pragmatic. This means instituting campaigns and interventions that can help Latina immigrant mothers navigate their new environment through the access and cooking of new healthy foods. For example, health providers and researchers can develop and implement campaigns/interventions that show Latina mothers what meals can be prepared with foods provided by WIC. For instance, providing hands-on interventions by cooking easy meals using WIC foods can have important health benefits among this population. Moreover, if it is imperative to provide education to Latina immigrant women, then it should focus on providing examples about which foods contain which types of nutrients.

### Responsiveness to family needs and wants as determinants of food choices

In this study it also became clear that ensuring the wellbeing of the family was a prominent determining factor in mothers’ decisions about what to feed their children. Not surprisingly, the participants in this study stated that they had changed their eating patterns during pregnancy or after having children in order to promote their health and wellbeing. Previous studies had found similar results regarding the importance of children and family [21, 28, 30, 33]. For instance, a study among Latina immigrant women (n = 36) found that husband’s food preferences were important when deciding what food to prepare [33]. This shows that family plays an important role when making decisions around food. In this study we discovered that the importance of family starts as early as pregnancy and remains over time. Overall, mothers are willing to change long-held behaviors in order to make sure their children eat a healthy and nutritious diet. This suggests that pregnancy and childrearing stages provide a perfect window of opportunity for intervention and promotion of healthy behaviors. During these periods, participants can be more receptive to advice and they might be more willing to change to make sure that they are doing the best they can to have a healthy family.

### Intrapersonal conflict stemming from childhood poverty and food insufficiency

Finally, in this study we found that mothers may experience internal conflict when deciding what and how to feed their children. We found that Latina mothers may have disagreements with their children over food because they tend to be more used to American foods. This study supports previous research findings that have demonstrated that intergenerational disagreements between mothers and children arise over food [18, 20]. Some of this research has claimed that Latino parents might engage in an indulgent feeding style [20]; however, the reasons behind this feeding style have not been explored. Our study adds to this knowledge by presenting information regarding the intrapersonal conflict that low-income Latina mothers engage in when trying to decide what and how to feed their children. Not only do mothers need to resist succumbing to their children’s pressure of wanting to eat certain foods, but they also need to resist the pressure to give in that arises from the scarcity they faced during childhood. Latina immigrant mothers find themselves in a difficult conundrum; they either let their children dictate what to eat, or they deprive their children from their favorite foods, even though they might not be able to afford them.

Contrary to other tensions that Latina immigrant women experience when deciding what to feed their children, this particular tension might be the most difficult to manage. Health and social service providers can play a role in easing this tension by acknowledging the fear low-income Latina mothers might have in relation to food scarcity and hunger. Providers can help clients reframe these thoughts by empowering them and reminding them that even small increases in resources, such as WIC benefits, can increase their capacity to offer their children healthy, nutritious food.

## Strengths and limitations

This study has multiple strengths including the use of the socio-ecological model to illustrate the tensions that Latina immigrant mother experience across and between levels of their social ecology when deciding what and how to feed their children. Also, this study highlights the experiences of women from different Latin American countries residing in non-metropolitan and small metro communities. Moreover, the data collection and analysis were conducted in Spanish by a bilingual and bicultural team, which enhanced the cultural sensitivity and interpretation of findings. Despite these strengths, this study is not without limitations. The participants interviewed in this study were all part of the WIC program and were under 185% of the Federal Poverty Level. Although participants from different nationalities were included in our study, our sample includes a particular set of Latinos. Most of our participants are low-income and grew up in extreme poverty. In fact, the majority mentioned that the reason they migrated to the U.S. was for economic reasons; in search of better economic opportunities. Because of these specific characteristics, the results of this study cannot be generalized to other Latino immigrant populations who grew up with more resources and income, or those residing in large metropolitan areas. Another limitation is that we did not collect information on mother or child health status. However, some participants volunteered some of this information as they were describing their feeding practices. A few participants mentioned that they changed their feeding practices when they found out they had certain chronic health condition such as diabetes. Future studies investigating eating practices should gather information on health status to try to determine the role that chronic health conditions play in relation to eating and feeding practices.

## Conclusion

When deciding what to feed their children, Latina immigrant mothers engage in a complex and multilayered process. We used a socioecological model to explain the conflicts that mothers engage in at different levels when trying to decide what to feed their children. We found that although Latina immigrant mothers want to feed their children healthy and nutritious foods, there are certain tensions that make it difficult to do so. We recommend that when an obesity intervention is developed for Latino immigrants, their base knowledge be valued and promoted. Finally, healthy eating interventions should take into account the many ways in which Latina mothers’ care and concern for family drives decision-making and can motivate positive health behavior change.

## Supporting information

S1 File Interview guide in English. English interview guide used in semi-structured interview.
(DOCX)
