# Supplementary material for: Feeding decision-making among first generation Latinas living in non-metropolitan and small metro areas
Source: PLoS One. 2019 Mar 18;14(3):e0213442. doi: 10.1371/journal.pone.0213442 (PMC6422285; doi:10.1371/journal.pone.0213442)
Supplement: S1 File — English interview guide used in semi-structured interview. (DOCX) [file pone.0213442.s001.docx]

Interview protocol

First and foremost I would like to thank you for agreeing to participate in this interview. Again, I would like to remind you that everything we discuss during this interview will remain completely private and confidential. Any information that we share is going to stay between us and I am not going to share it with anyone else and if I do, I will change your name and any other type of information that can help identify you. The reason I am doing this interviews is because I would like to understand a little bit more about eating patterns among Latino families and especially understand how mothers decide what to feed their children. Any information that you can share with me is essential and it would really help me understand more about eating patterns in our culture. Your participation is completely voluntary and if there is any question that you do not feel comfortable answering you just need to tell me and we will move on to the next question. Also, if there are any questions that are not very clear do not hesitate to ask me for an example or to ask me what I mean by that question.

First of all I would like you to tell me a little bit about your life in your home country and your migration process to the United States.

- How long ago did you move to the US
- What were some of the main reasons you decided to come?
- When you came to the US which city did you go to?
- What was it like for you when you first came here?
- What has it been like for you after having spent some years in the US?
- Would you like to go back to your home country at some point?
- What are some of the things that you miss the most about your country?

Thank you very much for your information. Like I was saying before, one of the topics that I am highly interested about is the food that you and your family consume. First of all, could you describe your family and who makes part of it?

- Who lives in the house?
- How many children do you have?
- What are their ages?

Now, could you tell me a little bit about the meals that you have at home?

- Who is in charge of grocery shopping?
- Usually who prepares the food?
- Can you describe a typical breakfast? a lunch? A dinner?
  - Usually what food do you serve during these meals?
- Do you have any type of routines before cooking?
- Where does everyone usually sit to eat?
  - Do you eat together or separate?
- What do you usually talk about during meals when everyone sits together?

Now I would like you to think a little bit about the meals that you used to have in your country of origin with your family. Can you describe any of these family meals?

- What did you usually eat?
- Was there any type of rules in your house when it came to food?
  - For example, did you have to finish all the food before leaving the table or something similar?
- Do you use any of these rules with your children?
- Which family members were present when you ate?
- How do you think that the food with which you feed your children is influenced by what you used to eat when you were little?
- Has anything changed in terms of your cooking because of your partner?
  - Or because of your children?
- When you started to feed your most recent baby, who did you talk to in order to find out what to feed him/her?

Now I would like to ask you a little bit about your last pregnancy and the food you ate during that time.

- How was your pregnancy like?
- How did you feel during the first year of life of your most recent baby?
- After the pregnancy were you satisfied with your weight?
  - Or were you preoccupied?
- Did you feel morning sickness or nausea during your last pregnancy?
  - Or did you have any other complications?
- Was there any type of food that you were craving for during your pregnancy?

Usually during pregnancy and also afterwards there are many women who feel a little down or nervous. This is quite normal, especially among immigrant mothers given that we do not have our families close by supporting us.

- During pregnancy or afterwards did you feel sad?
  - Or did you feel nervous?
- At any moment, not necessarily during pregnancy, have you felt sad?
- When you felt this way did you notice any changes in your feeding patterns?
  - Did you tend to eat more or less?
  - What type of things do you tend to eat when you feel sad?
- Did you ever notice this type of changes in your mom?
  - Or anybody else in your family?
  - Or among your friends?
- Finally, when you feel sad or nervous who do you talk to?
  - Do you call home?
  - Do you talk to your friends?
  - Or do you visit a psychologist (specialist)?

Those were all the questions I had for you. Again, thank you very much for all your help. Is there anything that you would like to add or tell me?

Lastly, do you know any other moms that you think would be interested in participating? I am looking for moms that have a child under the age of 2 and who have migrated to the US from another country. This is my information in case you would like to recommend someone.
